# The Association of Social and Biological Factors with Clinical Outcomes in Patients with Lung Cancer and DNA Damage Repair Pathway Mutations: A Single-Institution Experience

**DOI:** 10.3390/cancers18162606

**Published:** 2026-08-13

**Authors:** Ahmad Nanaa, Jeremy Kao, Martin Davis, Mary Pasquinelli, Margaret Wright Geise, Li Liu, Ryan Huu-Tuan Nguyen, Frank Weinberg

**Affiliations:** 1 Department of Medicine, University of Illinois Chicago, Chicago, IL 60607, USAmdavis48@uic.edu (M.D.); mpasqu3@uic.edu (M.P.); liliu@uic.edu (L.L.); rnguye8@uic.edu (R.H.-T.N.); 2 Data Integration Shared Resource, University of Illinois Cancer Center, Chicago, IL 60612, USA; mewright@uic.edu

**Keywords:** DNA damage response (DDR), socioeconomic status (SES), *ATR*, lung cancer, survival

## Abstract

**Background:** Lung cancer remains the leading cause of cancer-related mortality in the United States, characterized by poor overall survival rates (OS), particularly for advanced-stage disease. While smoking is a primary risk factor, other contributors include environmental exposures, genetics, comorbidities, and socioeconomic factors. **Methods:** We included 232 patients with lung cancer (96% NSCLC). Patients were categorized into DNA damage response (DDR)-mutant (DDR^mt^, *n* = 67, 29%) and DDR-wild-type (DDR^wt^, n = 165, 71%) groups. We evaluated the correlations between individual and socioeconomic factors between DDR^mt^ and DDR^wt^ lung cancer patients. **Results:**Nineteen DDR genes were identified, with *ARID1A* *(*31.3%*)*, *CHEK2 (*19.4%*)*, *ATM (*14.9%*)*, *BRCA2 (*14.9%*)*, *ATRX (*11.9%*)*, *MUTYH (*11.9%*)*, *ATR (*8.9%*)*, and *MSH6* (5.9%) being the most common. The DDR^mt^ group had a significantly higher median tumor mutational burden (TMB) (12 vs. 9; *p* = 0.006) and a higher prevalence of adenocarcinoma (85.1% vs. 64.8%, *p* = 0.003). Logistic regression identified adenocarcinoma histology (OR = 10.03, *p* = 0.002) and lower area deprivation index (OR = 0.98, *p* = 0.04) as predictors of DDR mutation status. Having advanced stage at diagnosis was associated with, age (*p* = 0.03), lack of lung cancer screening (*p* < 0.001), and adenocarcinoma histology (*p* < 0.001). While median OS was 61 months for the DDR^mt^ group vs. 44 months for the DDR^wt^ group (*p* = 0.654), patients with *ATR* mutations had significantly shorter survival (13 vs. 67 months; *p* = 0.0008). Population-level factors, including food insecurity (HR = 2.33, *p* = 0.002), lack of insurance (HR = 2.39, *p* = 0.008), and proximity to potential chemical accidents (HR = 1.73, *p* = 0.037), were significant predictors of OS. **Conclusions:** These findings suggest that DDR^mt^ lung cancers represent a biologically distinct subgroup characterized by higher tumor mutational burden and enrichment for adenocarcinoma histology; however, DDR mutation status alone was not associated with overall survival. Instead, outcomes appeared to vary by individual DDR gene, with *ATR* alterations identifying a subgroup with particularly poor survival. In parallel, food insecurity, lack of insurance, and proximity to potential environmental hazards were associated with outcomes, highlighting the need to integrate genomic biomarkers with social and environmental determinants of health. These data suggest the need for future prospective studies incorporating treatment-response data, longitudinal social determinants of health (SDOH) assessment, and environmental exposure measures to define how DDR alterations can guide precision oncology strategies while addressing modifiable barriers to equitable cancer care.

## Simple Summary

Lung cancer remains a leading cause of cancer-related mortality, prompting researchers to investigate how a combination of patient factors, molecular profiles, and environmental conditions influence outcomes. This work explores how genetic mutations in DNA repair mechanisms (DDR), alongside socioeconomic factors like food security and insurance status, impact cancer characteristics and survival (OS). We aim to identify distinct subgroups of lung cancer that may require more tailored treatment approaches. Our findings indicate that DDR-mutant lung cancer had higher tumor mutational burden, and a higher prevalence of adenocarcinoma histology. While DDR mutations do not predict OS, specific *ATR* alterations correlate with shorter OS. Furthermore, factors such as food insecurity, lack of insurance, and proximity to environmental hazards are independent predictors of outcomes. This study emphasizes the value of integrating genomic data with social determinants of health to advance precision medicine and ensure more equitable cancer care.

## 1. Introduction

Lung cancer remains the leading cause of cancer-related mortality in the United States, imposing a significant burden on both patients and the healthcare system. Despite continuous advancements in screening, staging, and the delivery of multimodal therapies, lung cancer places 6th lowest in terms of overall survival rates (OS) among all cancers globally, with a 5-year survival rate between 3 and 9% for advanced-stage disease [1,2,3,4,5].

Tobacco smoking remains the number one risk factor; notably, 75–90% of all lung cancer cases are smoking-related. Approximately 25% of all lung cancer cases are not associated with smoking with the vast majority classified as non-small cell lung cancer (NSCLC) [6,7]. Other environmental and behavioral risk factors include exposure to second-hand smoke, biomass fuels, arsenic, radon, asbestos, and diesel exhaust [8]. Furthermore, individual susceptibility is influenced by genetic predisposition—such as having a first-degree relative with the disease—and chronic comorbidities, including chronic obstructive pulmonary disease (COPD) and human immunodeficiency virus (HIV) [9,10,11,12].

Health outcomes are further mediated by profound socioeconomic disparities. In the United States, African American (AA) patients experience a disproportionately higher disease burden compared to other racial and ethnic groups [5,13]. Access to healthcare and insurance coverage are critical determinants of these outcomes: patients with private insurance are more likely to undergo timely screening and receive an earlier diagnosis, whereas uninsured or Medicaid-insured populations face systemic barriers that often result in delayed care and worse overall survival [14,15,16,17].

The Area Deprivation Index (ADI)—a composite metric of income, education, and employment—further illustrates the relationship between socioeconomic status (SES) and health outcomes [18]. Patients living in more deprived areas have shorter OS and delays in cancer care [18,19,20,21,22,23,24,25]. It has been reported that socioeconomic status can impact the molecular signature pattern, including higher associations with *KRAS* mutations [23,26,27,28,29].

Genome instability, characterized by an elevated mutation rate is a hallmark of malignancy that promotes tumor evolution and clonal heterogeneity. While the DNA damage response (DDR) system functions to maintain genomic integrity, its dysregulation through mutations in pathways such as homologous recombination (HR) and non-homologous end joining (NHEJ) promote clonality and drive carcinogenesis. DNA damage response and repair (DDR) gene alterations have been associated with higher tumor mutational burden (TMB), genomic instability, and enhanced tumor-infiltrating lymphocytes [30]. However, the role of these alterations in predicting treatment response and prognosis in NSCLC without oncogenic drivers remains unclear. Platinum-based chemotherapy, a cornerstone in NSCLC treatment, is cytotoxic by inducing DNA damage that a functional DDR system would otherwise repair; consequently, DDR-deficient tumors may exhibit enhanced sensitivity to these agents [26,30,31,32,33,34,35].

Despite the well-established role of DDR pathways in other malignancies—where they have enabled the successful clinical application of poly (ADP-ribose) polymerase (PARP) inhibitors in breast, ovarian, pancreatic, and prostate cancer [30,32,33,35,36,37]. To date, clinical evaluations of PARP inhibitors in non-small cell lung cancer (NSCLC) have yet to establish a definitive therapeutic benefit in routine practice. PARP inhibitors have demonstrated limited efficacy in lung cancers with suspected homologous recombination deficiency (HRD), and the association between DNA damage response (DDR) mutations and the efficacy of platinum-based chemotherapy in NSCLC remains controversial [37].

In this study, we investigate the landscape of DDR mutations in a cohort of lung cancer patients treated at an academic urban medical center serving an underserved population. We compared clinical outcomes between DDR-mutant (DDR^mt^) versus wild-type cases (DDR^wt^). We evaluated the correlations between individual and socioeconomic factors on DDR^mt^ and DDR^wt^ lung cancer patients.

## 2. Methods

### 2.1. Study Design

This is a single-institution study at the University of Illinois Hospital (UIH). After Institutional Review Board (IRB) approval, we retrospectively screened for all lung cancer patients treated at the University of Illinois Hospital between 2 November 2015 and 11 September 2023 with Tempus genetic data available.

Review of the electronic medical record was conducted for all patients and various individual-level factors were collected (Supplemental Methods S1). The home addresses of each patient were geocoded to the census tract level and mapped using a standardized coordinate reference system (Supplemental Figures S1 and S2). Each patient was linked with certain population-level social determinants of health (SDOH) and cancer risk factors compiled by the University of Illinois Cancer Center Data Integration Shared Resource, as described (Supplemental Table S1).

Patients were stratified based on the presence (DDR^mt^) or absence (DDR^wt^) of a pathogenic or likely pathogenic somatic mutation in a DDR pathway gene. DNA sequencing (xT) and whole-transcriptome RNA sequencing (xR) from tumor specimens were performed by Tempus (Tempus AI, Inc, Chicago, IL, USA) [38,39,40,41]. DDR pathway genes were defined as the subset of those previously described. Neoantigen tumor burden was performed on all non-silent mutations as described by Beaubier et al. [38].

### 2.2. Statistical Analyses

Descriptive statistics were reported as counts (%) or medians (IQR). Comparisons of categorical parameters were performed using the Chi-square test (χ^2^). For continuous variables, *t*-test or Wilcoxon test was used. Overall survival (OS) estimates were calculated using Kaplan–Meier curves which were calculated from diagnosis date and the date of death due to any cause. Patients who were alive at the last follow-up date were censored at that time. The median duration of follow-up was calculated using the reverse Kaplan–Meier method. We conducted a retrospective survival analysis to evaluate the prognostic factors influencing patient outcomes. A Cox proportional hazards regression model was used to estimate hazard ratios (HR) and corresponding 95% confidence intervals (CI). To optimize the model and account for potential confounders, we utilized a backward selection strategy, retaining predictors that maintained statistical significance. The discriminatory performance of the final model was assessed using the concordance index (C-index), an overall C-index of >0.75, indicating a robust ability to discriminate between patient outcomes. Ordinal logistic regression was performed to identify correlations between stage at diagnosis and individual- and population-level factors. All analyses were conducted using R version 4.4.0. and JMP^®^ Student Edition Statistical significance was defined as *p* < 0.05.

## 3. Results

We identified a total of 232 patients with lung cancer, NSCLC (N = 222, 96%). Adenocarcinoma (N = 166, 75%), squamous cell carcinoma (N = 41, 18%), other (N = 15, 7%), and SCLC (N = 10, 4%). Of the 232 patients, 67 (29%) harbored at least one pathogenic mutations involving DDR pathway (DDR^mt^) and 165 (71%) were DDR^wt^ (N = 165, 71%) (Table 1).

**Figure 1:**
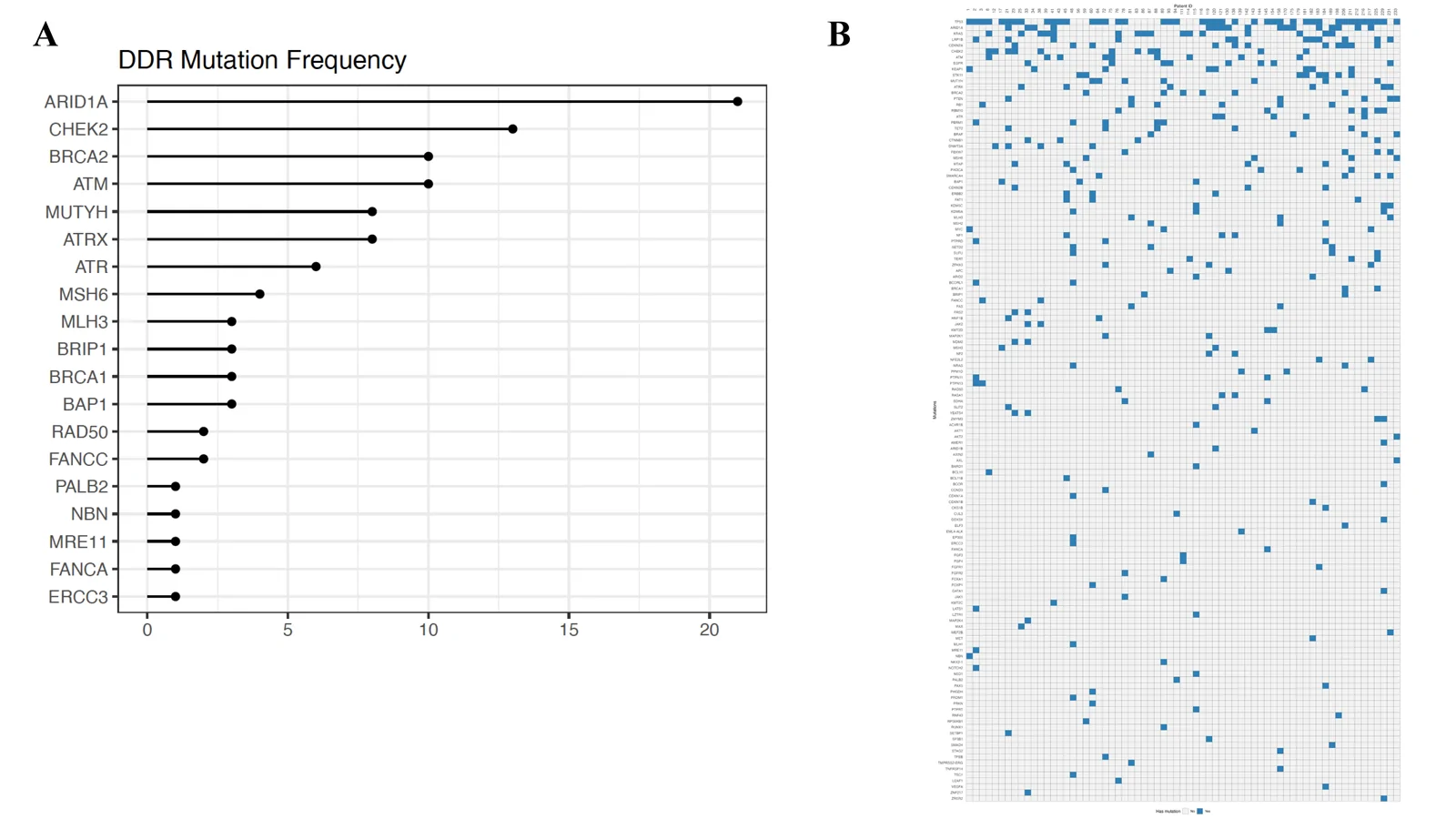
(**A**) Frequency of DNA damage repair (DDR) gene mutations. A total of 19 DDR pathway genes were identified: ARID1A, ATM, ATR, ATRX, BAP1, BRCA1, BRCA2, BRIP1, CHEK2, ERCC3, FANCA, FANCC, MLH3, MRE11, MSH6, MUTYH, NBN, PALB2, and RAD50. (**B**) Integrated mutation matrix mapping of DDR mutant lung cancer patients and concurrent mutations.

A total of 19 DDR pathway genes were identified: *ARID1A*, *ATM*, *ATR*, *ATRX*, *BAP1*, *BRCA1*, *BRCA2*, *BRIP1*, *CHEK2*, *ERCC3*, *FANCA*, *FANCC*, *MLH3*, *MRE11*, *MSH6*, *MUTYH*, *NBN*, *PALB2*, and *RAD50* (Figure 1).

Within the DDR^mt^ group, 53 (80%) patients harbored a single mutation in the DDR pathway and 14 (20%) had multiple mutations within the DDR-gene pathway. The most commonly mutated DDR genes were *ARID1A* (31.3%), *CHEK2* (19.4%), *ATM* (14.9%), *BRCA2* (14.9%), *ATRX* (11.9%), *MUTYH* (11.9%), *ATR* (8.9%), and *MSH6* (5.9%) (Figure 1).

Comparing the individual-level factors and population-level factors between the DDR^mt^ and the DDR^wt^ cohort. There was no significant difference between the two groups with respect to age, ethnicity, sex, insurance status, tobacco smoking status, number of pack years, having prior screening with LDCT, stage at diagnosis, or family history (*p* > 0.05). There was a significant difference between the two groups with respect to cancer type (*p* = 0.003), with the DDR^mt^ group having more patients with adenocarcinoma (85.1%) compared to the DDR^wt^ group (64.8%). The median tumor mutational burden (TMB) for the DDR^mt^ group was significantly higher than DDR^wt^ group (12 vs. 9; *p* = 0.006) (Supplemental Figure S3).

Regarding the population-level factors, there was no significant difference between the two groups with respect to ADI, binge drinking rate, those who visited their doctor for a routine checkup, hardship index, limited English proficiency, number of nearby federally qualified health centers, and severe housing cost burden (*p* > 0.05) (Table 1). There were no significant differences between the two groups with respect to any of the other population-level factors.

### 3.1. Predictors of DDR Mutational Status

A logistic regression analysis employing backward selection was performed to determine predictors of DDR^mt^ status across 225 observations. When using squamous cell carcinoma as the reference group, histological subtype significantly influenced the odds of being DDR^mt^. Patients with adenocarcinoma (OR = 10.03, CI 2.89–63.42, *p* = 0.002) or other category (OR = 7.52, CI 1.41–57.84, *p* = 0.026) had significantly higher odds of DDR^mt^ status compared to the reference) . The area deprivation index (OR = 0.98, CI 0.96–1.00, *p* = 0.04) was found to be also a significant predictor of DDR^mt^ status. PM2.5 concentration refers to particulate matter with a diameter of 2.5 micrometers or less, which are small inhalable particles that can penetrate deep into the lungs and deposit on the surface of the deeper parts of the lung [42]. While showing a positive trend, PM2.5 concentration did not reach statistical significance as a predictor for DDR^mt^ status (OR = 6.31, CI 1.00–53.38, *p* = 0.068) (Supplemental Table S2).

### 3.2. Predictors of Stage at Diagnosis

The analysis of individual-level factors (Table 2) identified three variables significantly associated with the stage at diagnosis. Age at diagnosis (*p* = 0.03), not having lung cancer screening (LDCT) completed (*p* < 0.001), and histological subtype of adenocarcinoma (*p* < 0.001) were significantly associated with a higher stage at diagnosis. There was no association between stage at diagnosis with respect to ethnicity, sex, insurance type, tobacco use, DDR mutation status, or having family history of lung cancer (*p* > 0.05).

In the overall cohort, utilizing a 75th percentile cutoff to separate the population-level factors into “low” and “high” categories. We found a statistically significant difference in the median age (*p* = 0.048), limited English proficiency (*p* = 0.031), low food access (*p* = 0.05), and social vulnerability index (*p* = 0.026) as a predictor for stage at diagnosis (Table 3).

### 3.3. Predictors of Survival

**Figure 2:**
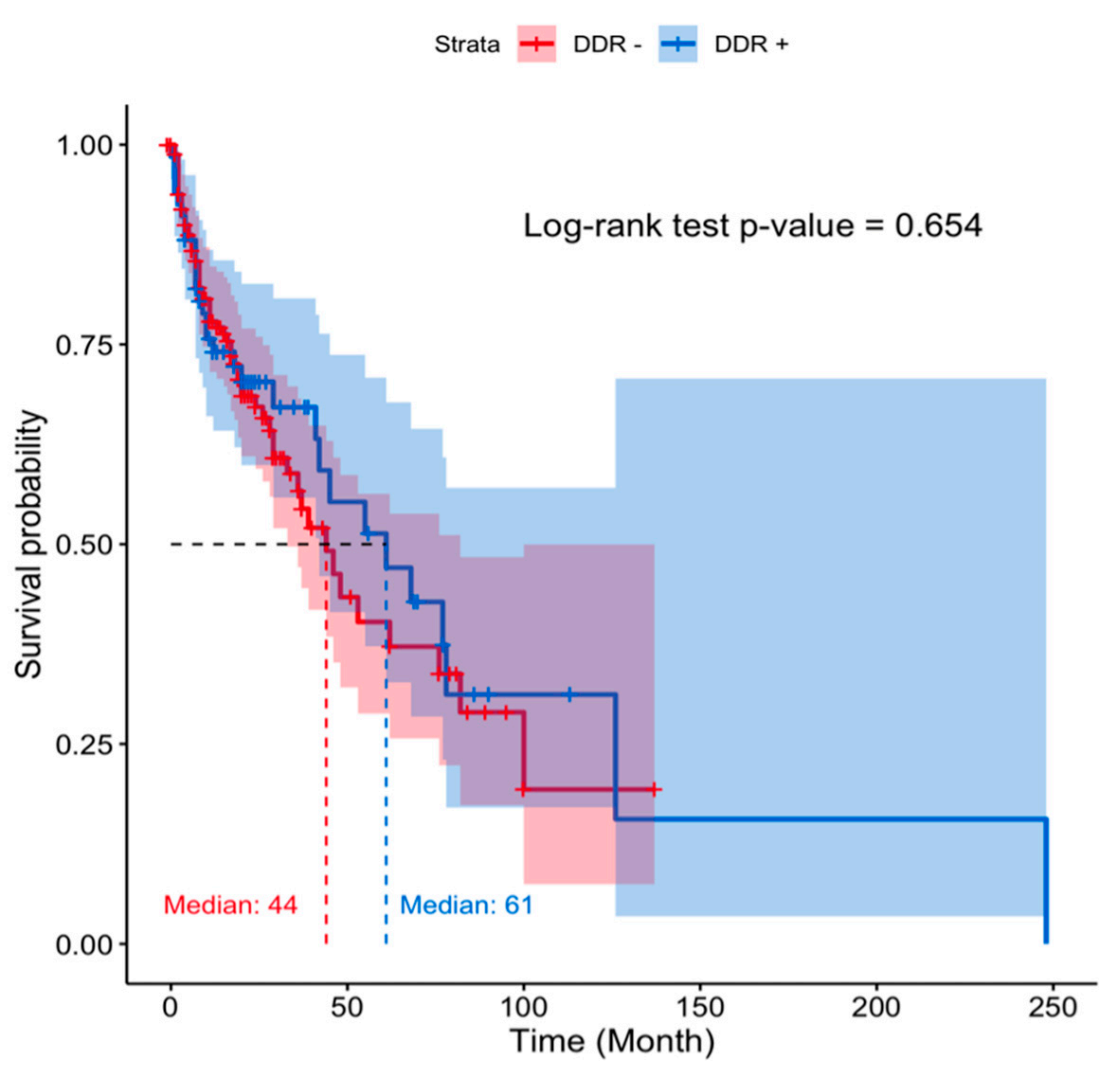
Kaplan–Meier plot of overall survival by DDR status.

Comparing median overall survival (OS), after a median follow-up of 23 months [95% CI: 20–27]. 14 patients with incomplete survival data were excluded, resulting in N = 220. DDR^mt^ patients had longer median OS of 61 months vs. 44 months in the DDR^wt^ patients; however, this was not statistically significant (*p* = 0.654) (Figure 2).

Individual-level factors associated with worse OS were higher stage at diagnosis (HR = 1.70, CI 1.34–2.17, *p* < 0.001), and younger age (<60 years) (HR = 2.85, CI 1.53–5.31, *p* = 0.001), compared to patients aged ≥ 60. Having a cancer type of “other” (HR = 3.15, CI 1.30–7.61, *p* = 0.011) compared to squamous cell carcinoma and being an active tobacco smoker (HR = 3.89, CI 1.61–9.40, *p* = 0.003) or a former smoker (HR = 2.30, CI 1.02–5.19, *p* = 0.044) relative to never-smokers were associated with significantly higher HR. An insurance type of “other” (HR = 7.22, CI 1.64–31.76, *p* = 0.009) was associated with significantly higher HR compared to private insurance status; however, there was no statistically significant difference based on insurance type (Medicaid or Medicare; *p* > 0.05) compared to private insurance. There was no significant difference in survival based on DDR mutational status (HR = 1.16, CI 0.72–1.87, *p* = 0.553) (Table 4).

**Figure 3:**
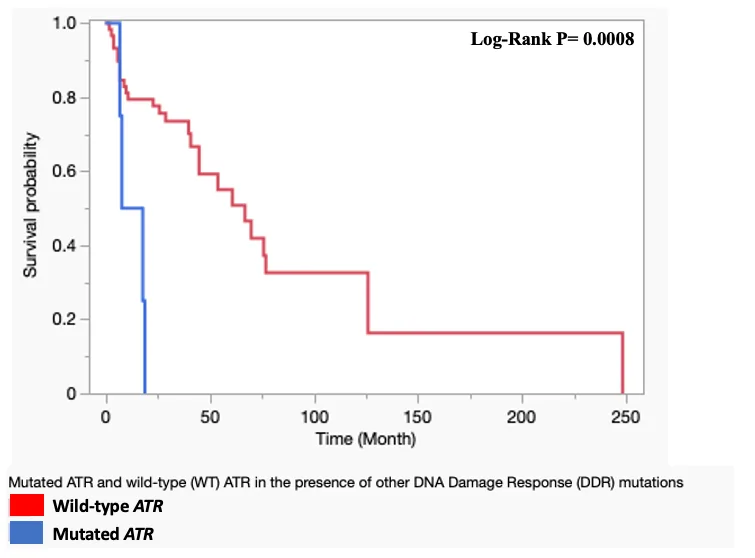
Kaplan–Meier plot of overall survival by ATR status. Mutated ATR and wild-type ATR in the presence of other DNA damage response (DDR) mutations.

Within the DDR^mt^ cohort, the median OS was significantly shorter for patients with mutated *ATR* gene (mOS 13 vs. 67 months; *p* = 0.0008), compared to *ATR* wild-type (Figure 3) all 4 patients with mutated *ATR* experienced death. There was no statistically significant difference in OS when comparing patients harboring mutations in the most commonly mutated DDR genes (*ARID1A*, *CHEK2*, *ATM*, *BRCA2*, *ATRX*, *MUTYH*, and *MSH6*) (*p* > 0.05) to their respective non-mutated counterparts. There was no statistically significant difference in OS comparing patients with single DDR vs. multiple DDR mutations (*p* > 0.05) in the DDR^mt^ cohort (Supplemental Figure S4).

For population-level factors, a 75th percentile cutoff was used to separate these population-level factors into “low” and “high” categories. Survival analysis to compare “low” and “high” categories of each population-level factor was performed. There was significantly worse OS in patients living in neighborhoods with lower food insecurity (HR = 2.33, CI 1.35–4.03, *p* = 0.002), lower uninsured rate (HR = 2.39, CI 1.26–4.54, *p* = 0.008), and a higher proximity to potential chemical accidents (HR = 1.73, CI 1.03–2.90, *p* = 0.037) (Table 5).

## 4. Discussion

In this single-institution cohort of patients with lung cancer undergoing comprehensive genomic profiling, 29% harbored at least one pathogenic or likely pathogenic alteration in a DDR pathway gene. DDR^mt^ tumors were enriched for adenocarcinoma histology and had significantly higher tumor mutational burden compared with DDR^wt^ tumors, consistent with prior reports linking DDR pathway alterations to genomic instability and increased tumor immunogenicity [43,44]. However, DDR mutation status alone was not associated with overall survival, suggesting that a binary DDR^mt^ versus DDR^wt^ classification may be insufficient to capture the biological and clinical heterogeneity of DDR-altered lung cancer.

The frequency of DDR alterations observed in our cohort is broadly consistent with prior studies, although estimates vary depending on tumor type, sequencing platform, gene panel composition, and definitions used to classify DDR alterations. Prior studies have reported DDR alteration rates ranging from approximately 14% to more than 30% in NSCLC and other solid tumors, with variability across histologic subtypes and DDR pathway categories [43,44,45,46]. In our cohort, the most frequently altered DDR genes were *ARID1A* (7%), *CHEK2* (5%), *ATM* (4%), *BRCA2* (3%), *ATRX* (3%), *MUTYH* (3%), *ATR* (2%), and *MSH6* (2%), which overlaps with previously reported DDR landscapes in advanced NSCLC [43]. Most patients harbored a single DDR alteration, while a smaller subset had multiple DDR pathway alterations, similar to prior reports [43]. These findings support the concept that DDR alterations are common in lung cancer, but also emphasize that DDR^mt^ tumors represent a heterogeneous group rather than a single clinically uniform entity.

Consistent with prior studies, DDR^mt^ tumors in our cohort had higher median TMB than DDR^wt^ tumors. This association is biologically plausible because impaired DNA damage recognition or repair can increase the accumulation of somatic alterations, potentially generating more neoantigens and altering tumor–immune interactions [43,44]. Prior studies have suggested that DDR alterations may be associated with improved response to PD-(L)1 blockade and platinum-based chemotherapy in selected NSCLC populations, likely reflecting both increased genomic instability and impaired repair of treatment-induced DNA damage [44]. In our cohort, DDR^mt^ patients had numerically longer median overall survival compared with DDR^wt^ patients, although this difference was not statistically significant. This may reflect limited sample size, treatment heterogeneity, variable co-mutational context, and the inclusion of multiple DDR genes with distinct functional consequences. Future studies should therefore evaluate DDR alterations not only as a single grouped biomarker, but also by pathway, gene, zygosity, functional impact, and interaction with TMB, PD-L1 expression, oncogenic drivers, and treatment exposure.

A particularly notable finding was the association between *ATR* mutation and shorter overall survival within the DDR^mt^ cohort. *ATR* is a central regulator of replication stress response and cell-cycle checkpoint signaling, and *ATR* pathway dysfunction may identify tumors with distinct biology, increased genomic instability, or greater therapeutic vulnerability [47,48]. Prior studies have reported *ATR* alterations in familial lung cancer and have also linked high *ATR* expression with worse prognosis in *KRAS*-mutated NSCLC [48]. In our cohort, patients with *ATR*-mutant tumors had markedly shorter survival compared with *ATR*-wild-type DDR-mutant patients. Although this finding should be interpreted cautiously given the small number of *ATR*-mutant cases, it raises the possibility that specific DDR genes, rather than overall DDR status, may define clinically meaningful risk groups. If validated, *ATR*-altered lung cancer could represent a rational subgroup for prospective studies evaluating intensified surveillance, platinum sensitivity, immunotherapy combinations, or DDR-targeted strategies such as *ATR*, PARP, or DNA-PK pathway inhibition.

This study also highlights the importance of integrating social and environmental determinants of health into precision oncology research. In univariate comparisons, DDR^mt^ and the DDR^wt^ cohort, we found no difference in outcomes (median survival time, stage at diagnosis), demographics (age, ethnicity, sex), individual socioeconomic factors (insurance status), or individual risk factors (smoking status, number of pack years, LDCT completed, family history) between the two groups (*p* > 0.05). DDR^mt^ and the DDR^wt^ patients did not differ significantly across most population-level SDOH variables. This may reflect the shared catchment area of our institution, which serves a predominantly urban population from communities facing overlapping socioeconomic and environmental challenges. However, in multivariable modeling, lower area deprivation index was associated with DDR mutation status, while PM2.5 exposure showed a nonsignificant trend toward association with DDR^mt^ disease. These findings should not be interpreted as causal, but they suggest that tumor genomic patterns may be shaped by a complex interaction of inherited susceptibility, environmental exposures, tobacco exposure, access to early detection, selection for molecular testing, and neighborhood-level conditions.

The potential relationship between SDOH and tumor genomics is supported by emerging literature across cancer types. In NSCLC, neighborhood disadvantage has been associated with risk of *KRAS*-mutated disease, suggesting that social context and environmental exposures may correlate with specific molecular tumor subtypes [19,20,21,22,23,26]. In breast cancer, studies have reported associations between household income and *TP53* mutation frequency, as well as differential DDR gene-expression patterns among Black women compared with White women [27,28]. In gastric cancer, socioeconomic disparities have also been associated with differences in the tumor genomic landscape [29]. Together, these studies support the hypothesis that social and environmental exposures may leave measurable biological imprints on tumor development, although the mechanisms remain incompletely defined. Potential pathways include chronic exposure to air pollution and inhaled carcinogens, oxidative stress, inflammation, tobacco-related DNA damage, nutritional deprivation, chronic psychosocial stress, and differences in comorbid disease burden or access to preventive care.

In the overall cohort, several population-level and individual-level factors were associated with survival, including food insecurity, lack of insurance, and proximity to potential chemical accident hazards. These findings reinforce the idea that precision oncology outcomes are not determined by molecular biomarkers alone. Food insecurity, for example, may not directly cause DDR alterations, but it may influence survival through multiple downstream mechanisms, including delayed care, competing financial needs, reduced ability to attend appointments, treatment interruptions, nutritional vulnerability, and decreased tolerance of systemic therapy. Lack of insurance may similarly affect access to screening, timely diagnosis, molecular testing, specialty oncology care, and receipt of guideline-concordant therapy. Proximity to potential chemical hazards may function both as a marker of environmental exposure and as a broader indicator of structural disadvantage. Thus, SDOH factors may influence lung cancer outcomes through both biological and care-delivery pathways.

These findings have several implications for future interventional studies. First, prospective DDR-focused lung cancer cohorts should incorporate structured SDOH assessment, environmental exposure mapping, treatment-response data, and longitudinal survival outcomes. This would allow investigators to determine whether DDR alterations predict response to platinum chemotherapy, immunotherapy, or DDR-directed agents after accounting for social vulnerability and access-to-care factors. Second, clinical trials evaluating DDR-directed therapies should consider stratifying or adjusting for key SDOH variables to avoid attributing outcome differences solely to tumor biology. Third, pragmatic precision-oncology interventions should test whether genomic testing combined with patient navigation, insurance support, food assistance, smoking cessation, transportation support, and environmental-risk-informed outreach can improve both trial participation and real-world outcomes. This integrated approach may be particularly important in safety-net settings, where molecular vulnerability and social vulnerability frequently coexist.

Our study has several limitations. First, the retrospective, single-center design restricts the generalizability of our findings to broader, multi-institutional patient populations. The inclusion criteria were restricted to patients who underwent Tempus genomic profiling, which introduces a potential selection bias; these patients may not be representative of the general oncology population, as genomic testing utilization often correlates with specific socioeconomic factors and disease stage. Our sample size was modest, which limited our statistical power, increasing the risk of overfitting in our multivariable models and precluding granular subgroup analyses. We also categorized DDR genes heterogeneously, which fails to account for the distinct biological functions and clinical implications of specific DDR pathways, co-mutational patterns, and treatment-specific outcomes. We were also unable to stratify DDR^mt^ and DDR^wt^ groups by the presence of actionable oncogenic drivers, which may influence prognosis and treatment selection. In addition, population-level SDOH variables were assigned based on geocoded residential location and therefore may not fully capture individual-level lived experience, including household food insecurity, income, transportation barriers, social support, or financial strain. Finally, the association between SDOH variables and DDR alterations should be considered hypothesis-generating and requires validation in larger prospective cohorts.

## 5. Conclusions

In conclusion, DDR pathway alterations are common in lung cancer and may be associated with higher TMB and adenocarcinoma histology, though DDR mutation status alone did not predict overall survival in this cohort. Our exploratory findings raise the hypothesis that specific DDR genes, particularly *ATR*, may have distinct prognostic implications; however, these observations are based on small subgroup numbers and a heterogeneous DDR classification scheme, and therefore should be interpreted with caution. Similarly, area-level social and environmental determinants of health were associated with survival differences, though the reliance on population-level SDOH measures rather than individual-level data limits the strength of these conclusions. Given the retrospective, single-center design and the potential for selection bias, these results should be considered hypothesis-generating. Confirmation in larger, prospective, multi-institutional cohorts is needed to validate these findings. If confirmed, integrating genomic biomarkers, environmental exposure data, and individual-level SDOH measures may help inform biologically driven and equity-focused strategies for improving lung cancer outcomes.

## Figures and Tables

**Table 1:** Clinical characteristics of 232 patients with lung cancer by DDR status.

	DDR − (*N* = 165)	DDR + (*N* = 67)	*p*-Value
**Survival Time (Month)**			
Median	44	61	0.654 ^[3]^
95% CI	(33–82)	(42–NA)	
**Age**			
<60	34 (20.6%)	11 (16.4%)	0.562 ^[1]^
≥60	127 (77.0%)	55 (82.1%)	
Missing	4 (2.4%)	1 (1.5%)	
**Ethnicity**			
Hispanic	15 (9.1%)	9 (13.4%)	0.433 ^[1]^
Non-Hispanic Black	107 (64.8%)	38 (56.7%)	
Non-Hispanic White	22 (13.3%)	13 (19.4%)	
Others	21 (12.7%)	7 (10.4%)	
**Sex**			
Male	80 (48.5%)	31 (46.3%)	0.872 ^[1]^
Female	85 (51.5%)	36 (53.7%)	
**Insurance**			
Private Insurance	13 (7.9%)	9 (13.4%)	0.459 ^[1]^
Medicaid	63 (38.2%)	20 (29.9%)	
Medicare	86 (52.1%)	37 (55.2%)	
Others	3 (1.8%)	1 (1.5%)	
**Tobacco**			
Active	45 (27.3%)	23 (34.3%)	0.447 ^[1]^
Former	101 (61.2%)	35 (52.2%)	
Never	19 (11.5%)	9 (13.4%)	
**Pack Years**			
≤15	28 (17.0%)	12 (17.9%)	0.919 ^[1]^
>15	113 (68.5%)	45 (67.2%)	
Never	19 (11.5%)	9 (13.4%)	
Missing	5 (3.0%)	1 (1.5%)	
**LDCT Completed**			
No	127 (77.0%)	53 (79.1%)	0.919 ^[1]^
Yes	37 (22.4%)	14 (20.9%)	
Missing	1 (0.6%)	0 (0%)	
**Stage**			
I	30 (18.2%)	12 (17.9%)	0.285 ^[1]^
II	16 (9.7%)	8 (11.9%)	
III	40 (24.2%)	9 (13.4%)	
IV	75 (45.5%)	37 (55.2%)	
Missing	4 (2.4%)	1 (1.5%)	
**Cancer Type**			
Squamous Cell Carcinoma	39 (23.6%)	2 (3.0%)	0.003 ^[1]^
Adenocarcinoma	107 (64.8%)	57 (85.1%)	
Small Cell Carcinoma	7 (4.2%)	3 (4.5%)	
Others	12 (7.3%)	5 (7.5%)	
**Family History**			
No	138 (83.6%)	57 (85.1%)	0.942 ^[1]^
Yes	27 (16.4%)	10 (14.9%)	
**Area Deprivation Index**			
Mean (SD)	55.0 (17.9)	52.1 (17.1)	0.249 ^[2]^
Median [Min, Max]	60.9 (14.6–87.4)	58.4 (14.7–87.4)	
**Median Age**			
Median [Min, Max]	67 (34–88)	67 (45–94)	0.521 ^[2]^
**Binge Drinking (%)**			
Mean (SD)	15.4 (2.65)	16.0 (2.72)	0.087 ^[2]^
Median [Min, Max]	14.7 (11.8–23.4)	16.2 (12.2–23.4)	
**Visited Doctor for Routine Checkup (%)**			
Mean (SD)	77.3 (3.86)	76.4 (3.94)	0.091 ^[2]^
Median [Min, Max]	77.3 (70.7–83.5)	75.7 (70.8–83.5)	
**Proximity to Potential Chemical Accidents**			
Mean (SD)	2.59 (2.09)	2.73 (1.99)	0.632 ^[2]^
Median [Min, Max]	1.91 (0.278–8.68)	2.31 (0.181–8.68)	
**Violent Crime (per 100,000)**			
Mean (SD)	1480 (796)	1310 (707)	0.138 ^[2]^
Median [Min, Max]	1380 (0–3140)	915 (142–3140)	
Missing	27 (16.4%)	10 (14.9%)	
**High School Graduation Rate (%)**			
Mean (SD)	83.7 (7.92)	83.2 (8.48)	0.692 ^[2]^
Median [Min, Max]	85.0 (61.9–97.1)	85.0 (61.4–96.6)	
**Spanish Primary Language (%)**			
Mean (SD)	20.0 (20.8)	24.3 (22.7)	0.182 ^[2]^
Median [Min, Max]	9.10 (1.38–79.8)	12.9 (2.81–72.4)	
**Limited English Proficiency Households (%)**			
Mean (SD)	4.77 (6.57)	5.69 (6.76)	0.343 ^[2]^
Median [Min, Max]	1.82 (0–24.2)	1.56 (0–24.2)	
**No Exercise (%)**			
Mean (SD)	33.7 (7.15)	32.7 (6.80)	0.329 ^[2]^
Median [Min, Max]	35.6 (15.5–44.5)	33.6 (15.5–44.5)	
**Food Insecurity (%)**			
Mean (SD)	17.0 (3.86)	16.1 (3.96)	0.105 ^[2]^
Median [Min, Max]	17.0 (10.1–24.9)	16.0 (7.60–24.5)	
Missing	1 (0.6%)	1 (1.5%)	
**Living in Food Deserts (%)**			
Mean (SD)	1.48 (3.82)	1.27 (3.33)	0.676 ^[2]^
Median [Min, Max]	0.00569 (0–25.0)	0.171 (0–25.0)	
**Foreign Born (%)**			
Mean (SD)	17.0 (13.0)	18.9 (12.3)	0.3 ^[2]^
Median [Min, Max]	13.2 (1.54–45.8)	16.9 (1.54–40.9)	
**Federally Qualified Health Centers**			
Mean (SD)	4.83 (3.14)	5.97 (4.17)	0.061 ^[2]^
Median [Min, Max]	4.00 (1.00–15.0)	5.00 (1.00–15.0)	
Missing	15 (9.1%)	8 (11.9%)	
**Severe Housing Cost Burden (%)**			
Mean (SD)	22.1 (6.57)	20.5 (6.08)	0.077 ^[2]^
Median [Min, Max]	21.4 (9.67–36.7)	19.1 (4.88–36.7)	
**Hardship Index**			
Mean (SD)	69.1 (21.9)	67.5 (22.7)	0.64 ^[2]^
Median [Min, Max]	78.0 (3.77–92.6)	71.7 (3.77–92.6)	
**Crowded Housing (%)**			
Mean (SD)	3.65 (1.99)	3.87 (2.05)	0.456 ^[2]^
Median [Min, Max]	3.02 (0.491–10.9)	3.16 (0.984–8.93)	
**Median Household Income ($)**			
Mean (SD)	52,700 (20,800)	55,900 (21,600)	0.308 ^[2]^
Median [Min, Max]	49,500 (53.0–126,000)	50,800 (55.0–126,000)	
**Life Expectancy (years)**			
Mean (SD)	75.8 (3.57)	76.4 (3.21)	0.216 ^[2]^
Median [Min, Max]	75.6 (68.3–82.0)	77.3 (70.0–82.8)	
**Low Food Access (%)**			
Mean (SD)	32.3 (20.9)	33.5 (22.3)	0.695 ^[2]^
Median [Min, Max]	30.6 (0–94.0)	24.5 (0–77.6)	
**No Computer or Smartphone (%)**			
Mean (SD)	9.23 (3.47)	8.88 (3.90)	0.523 ^[2]^
Median [Min, Max]	9.09 (1.19–16.2)	8.07 (1.19–16.2)	
**No Vehicle Available (%)**			
Mean (SD)	25.3 (10.9)	23.8 (10.8)	0.339 ^[2]^
Median [Min, Max]	26.4 (3.89–43.2)	24.1 (2.98–41.2)	
**Obesity (%)**			
Mean (SD)	37.2 (6.72)	36.3 (6.23)	0.332 ^[2]^
Median [Min, Max]	38.5 (24.4–46.8)	37.6 (24.4–46.8)	
**Non-Hispanic White (%)**			
Mean (SD)	20.4 (20.7)	22.1 (21.1)	0.573 ^[2]^
Median [Min, Max]	13.7 (1.07–84.4)	20.1 (1.07–78.1)	
**Non-Hispanic Black (%)**			
Mean (SD)	45.8 (33.1)	38.8 (32.0)	0.141 ^[2]^
Median [Min, Max]	46.5 (0.817–93.4)	24.8 (0.660–93.4)	
**Asian (%)**			
Mean (SD)	6.53 (10.3)	6.90 (10.1)	0.799 ^[2]^
Median [Min, Max]	1.34 (0.0587–35.4)	3.32 (0.0587–35.4)	
**Hispanic (%)**			
Mean (SD)	24.8 (24.4)	29.6 (26.7)	0.203 ^[2]^
Median [Min, Max]	12.0 (2.63–87.9)	15.7 (2.63–82.9)	
**PM2.5 Concentration (µg/m^3^)**			
Mean (SD)	10.1 (0.232)	10.1 (0.211)	0.42 ^[2]^
Median [Min, Max]	10.1 (8.85–10.4)	10.1 (9.22–10.4)	
**Poverty Rate (%)**			
Mean (SD)	21.6 (8.41)	19.9 (7.96)	0.159 ^[2]^
Median [Min, Max]	22.6 (5.06–35.9)	18.0 (4.86–35.9)	
**Food Stamps (%)**			
Mean (SD)	26.3 (12.4)	23.9 (11.0)	0.155 ^[2]^
Median [Min, Max]	27.7 (3.93–50.4)	22.6 (5.06–49.5)	
**Proximity to Superfund Sites**			
Mean (SD)	0.0868 (0.131)	0.0713 (0.0414)	0.173 ^[2]^
Median [Min, Max]	0.0525 (0.0171–1.42)	0.0532 (0.0283–0.220)	
**Social Vulnerability Index (%)**			
Mean (SD)	72.0 (15.8)	70.1 (17.4)	0.441 ^[2]^
Median [Min, Max]	75.7 (25.5–93.6)	74.2 (24.9–93.6)	
**Cigarette Smoking Rate (%)**			
Mean (SD)	17.7 (4.65)	17.0 (4.02)	0.242 ^[2]^
Median [Min, Max]	18.5 (8.00–27.1)	18.0 (8.00–27.1)	
**Traffic Intensity**			
Mean (SD)	1630 (1680)	1640 (1660)	0.988 ^[2]^
Median [Min, Max]	1080 (76.8–10,500)	1110 (217–10,500)	
**Proximity to Hazardous Waste Management Sites**			
Mean (SD)	8.19 (5.33)	8.76 (5.90)	0.498 ^[2]^
Median [Min, Max]	6.85 (0.0894–30.7)	8.86 (0.846–30.7)	
**Unemployment Rate (%)**			
Mean (SD)	11.4 (5.11)	10.8 (4.62)	0.367 ^[2]^
Median [Min, Max]	10.8 (2.74–20.7)	10.4 (3.30–20.7)	
**Uninsured Rate (%)**			
Mean (SD)	10.2 (4.02)	10.2 (4.13)	0.991 ^[2]^
Median [Min, Max]	9.37 (3.12–18.2)	9.21 (4.08–18.2)	
**Proximity to Water Polluting Sites**			
Mean (SD)	108 (298)	110 (331)	0.97 ^[2]^
Median [Min, Max]	8.85 (0.00116–2030)	12.4 (0.155–2030)	
**Lung cancer diagnosis rate (per 100,000)**			
Mean (SD)	75.3 (19.5)	70.7 (20.0)	0.117 ^[2]^
Median [Min, Max]	82.3 (31.4–115)	71.5 (31.4–102)	
Missing	1 (0.6%)	1 (1.5%)	

^[1]^: χ^2^ test ^[2]^: *t* test; ^[3]^: log-rank test.

**Table 2:** Predictors of stage at diagnosis: Individual-level factors.

	I (N = 42)	II (N = 23)	III (N = 47)	IV (N = 108)	*p*-Value
**Age**					
<60	2 (4.8%)	1 (4.3%)	9 (19.1%)	30 (27.8%)	0.003 ^[1]^
≥60	40 (95.2%)	22 (95.7%)	38 (80.9%)	78 (72.2%)	
**Ethnicity**					
Hispanic	6 (14.3%)	2 (8.7%)	4 (8.5%)	11 (10.2%)	0.699 ^[1]^
Non-Hispanic Black	27 (64.3%)	16 (69.6%)	27 (57.4%)	65 (60.2%)	
Non-Hispanic White	5 (11.9%)	1 (4.3%)	11 (23.4%)	17 (15.7%)	
Others	4 (9.5%)	4 (17.4%)	5 (10.6%)	15 (13.9%)	
**Sex**					
Male	13 (31.0%)	13 (56.5%)	23 (48.9%)	53 (49.1%)	0.144 ^[1]^
Female	29 (69.0%)	10 (43.5%)	24 (51.1%)	55 (50.9%)	
**Insurance**					
Private Insurance	3 (7.1%)	0 (0%)	4 (8.5%)	14 (13.0%)	0.299 ^[1]^
Medicaid	10 (23.8%)	10 (43.5%)	20 (42.6%)	40 (37.0%)	
Medicare	29 (69.0%)	13 (56.5%)	22 (46.8%)	52 (48.1%)	
Others	0 (0%)	0 (0%)	1 (2.1%)	2 (1.9%)	
**Tobacco**					
Active	16 (38.1%)	7 (30.4%)	12 (25.5%)	29 (26.9%)	0.813 ^[1]^
Former	20 (47.6%)	14 (60.9%)	29 (61.7%)	65 (60.2%)	
Never	6 (14.3%)	2 (8.7%)	6 (12.8%)	14 (13.0%)	
**LDCT Completed**					
No	22 (52.4%)	15 (65.2%)	33 (70.2%)	100 (92.6%)	<0.001 ^[1]^
Yes	20 (47.6%)	8 (34.8%)	14 (29.8%)	8 (7.4%)	
**DDR Mutation**					
DDR −	30 (71.4%)	16 (69.6%)	38 (80.9%)	72 (66.7%)	0.359 ^[1]^
DDR +	12 (28.6%)	7 (30.4%)	9 (19.1%)	36 (33.3%)	
**Cancer Type**					
Squamous Cell Carcinoma	4 (9.5%)	3 (13.0%)	19 (40.4%)	11 (10.2%)	<0.001 ^[1]^
Adenocarcinoma	35 (83.3%)	19 (82.6%)	23 (48.9%)	80 (74.1%)	
Small Cell Carcinoma	1 (2.4%)	1 (4.3%)	1 (2.1%)	6 (5.6%)	
Others	2 (4.8%)	0 (0%)	4 (8.5%)	11 (10.2%)	
**Family History**					
No	35 (83.3%)	19 (82.6%)	42 (89.4%)	89 (82.4%)	0.739 ^[1]^
Yes	7 (16.7%)	4 (17.4%)	5 (10.6%)	19 (17.6%)	

[1]: χ^2^ test.

**Table 3:** Community-level predictors of stage at diagnosis using 75th percentile cutoffs.

	I (N = 42)	II (N = 23)	III (N = 47)	IV (N = 108)	*p*-Value
**Area Deprivation Index**					
High	12 (28.6%)	3 (13.0%)	7 (14.9%)	27 (25.0%)	0.258 ^[1]^
Low	30 (71.4%)	20 (87.0%)	40 (85.1%)	81 (75.0%)	
**Median Age**					
High	9 (21.4%)	6 (26.1%)	12 (25.5%)	45 (41.7%)	0.048 ^[1]^
Low	33 (78.6%)	17 (73.9%)	35 (74.5%)	63 (58.3%)	
**Binge Drinking (%)**					
High	10 (23.8%)	5 (21.7%)	11 (23.4%)	31 (28.7%)	0.826 ^[1]^
Low	32 (76.2%)	18 (78.3%)	36 (76.6%)	77 (71.3%)	
**Visited Doctor for Routine Checkup (%)**					
High	9 (21.4%)	5 (21.7%)	11 (23.4%)	30 (27.8%)	0.82 ^[1]^
Low	33 (78.6%)	18 (78.3%)	36 (76.6%)	78 (72.2%)	
**Proximity to Potential Chemical Accidents**					
High	11 (26.2%)	4 (17.4%)	17 (36.2%)	27 (25.0%)	0.344 ^[1]^
Low	31 (73.8%)	19 (82.6%)	30 (63.8%)	81 (75.0%)	
**High School Graduation Rate (%)**					
High	7 (16.7%)	8 (34.8%)	10 (21.3%)	31 (28.7%)	0.284 ^[1]^
Low	35 (83.3%)	15 (65.2%)	37 (78.7%)	77 (71.3%)	
**Spanish Primary Language (%)**					
High	12 (28.6%)	7 (30.4%)	15 (31.9%)	20 (18.5%)	0.231 ^[1]^
Low	30 (71.4%)	16 (69.6%)	32 (68.1%)	88 (81.5%)	
**No Exercise (%)**					
High	14 (33.3%)	6 (26.1%)	15 (31.9%)	22 (20.4%)	0.282 ^[1]^
Low	28 (66.7%)	17 (73.9%)	32 (68.1%)	86 (79.6%)	
**Living in Food Deserts (%)**					
High	7 (16.7%)	6 (26.1%)	10 (21.3%)	36 (33.3%)	0.152 ^[1]^
Low	35 (83.3%)	17 (73.9%)	37 (78.7%)	72 (66.7%)	
**Foreign Born (%)**					
High	15 (35.7%)	8 (34.8%)	15 (31.9%)	27 (25.0%)	0.518 ^[1]^
Low	27 (64.3%)	15 (65.2%)	32 (68.1%)	81 (75.0%)	
**Severe Housing Cost Burden (%)**					
High	10 (23.8%)	5 (21.7%)	10 (21.3%)	21 (19.4%)	0.947 ^[1]^
Low	32 (76.2%)	18 (78.3%)	37 (78.7%)	87 (80.6%)	
**Hardship Index**					
High	17 (40.5%)	6 (26.1%)	16 (34.0%)	26 (24.1%)	0.208 ^[1]^
Low	25 (59.5%)	17 (73.9%)	31 (66.0%)	82 (75.9%)	
**Crowded Housing (%)**					
High	14 (33.3%)	8 (34.8%)	11 (23.4%)	21 (19.4%)	0.203 ^[1]^
Low	28 (66.7%)	15 (65.2%)	36 (76.6%)	87 (80.6%)	
**Median Household Income ($)**					
High	8 (19.0%)	5 (21.7%)	10 (21.3%)	37 (34.3%)	0.151 ^[1]^
Low	34 (81.0%)	18 (78.3%)	37 (78.7%)	71 (65.7%)	
**Limited English Proficiency Households (%)**					
High	10 (23.8%)	7 (30.4%)	11 (23.4%)	11 (10.2%)	0.031 ^[1]^
Low	32 (76.2%)	16 (69.6%)	36 (76.6%)	97 (89.8%)	
**Life Expectancy (years)**					
High	11 (26.2%)	5 (21.7%)	12 (25.5%)	31 (28.7%)	0.909 ^[1]^
Low	31 (73.8%)	18 (78.3%)	35 (74.5%)	77 (71.3%)	
**Low Food Access (%)**					
High	7 (16.7%)	4 (17.4%)	10 (21.3%)	38 (35.2%)	0.05 ^[1]^
Low	35 (83.3%)	19 (82.6%)	37 (78.7%)	70 (64.8%)	
**No Computer or Smartphone (%)**					
High	14 (33.3%)	7 (30.4%)	10 (21.3%)	23 (21.3%)	0.381 ^[1]^
Low	28 (66.7%)	16 (69.6%)	37 (78.7%)	85 (78.7%)	
**No Vehicle Available (%)**					
High	9 (21.4%)	6 (26.1%)	15 (31.9%)	21 (19.4%)	0.385 ^[1]^
Low	33 (78.6%)	17 (73.9%)	32 (68.1%)	87 (80.6%)	
**Obesity (%)**					
High	12 (28.6%)	3 (13.0%)	13 (27.7%)	30 (27.8%)	0.501 ^[1]^
Low	30 (71.4%)	20 (87.0%)	34 (72.3%)	78 (72.2%)	
**Non-Hispanic White (%)**					
High	9 (21.4%)	6 (26.1%)	12 (25.5%)	36 (33.3%)	0.47 ^[1]^
Low	33 (78.6%)	17 (73.9%)	35 (74.5%)	72 (66.7%)	
**Non-Hispanic Black (%)**					
High	12 (28.6%)	4 (17.4%)	13 (27.7%)	26 (24.1%)	0.748 ^[1]^
Low	30 (71.4%)	19 (82.6%)	34 (72.3%)	82 (75.9%)	
**Asian (%)**					
High	7 (16.7%)	4 (17.4%)	14 (29.8%)	29 (26.9%)	0.384 ^[1]^
Low	35 (83.3%)	19 (82.6%)	33 (70.2%)	79 (73.1%)	
**Hispanic (%)**					
High	12 (28.6%)	7 (30.4%)	15 (31.9%)	20 (18.5%)	0.231 ^[1]^
Low	30 (71.4%)	16 (69.6%)	32 (68.1%)	88 (81.5%)	
**PM2.5 Concentration (µg/m^3^)**					
High	12 (28.6%)	6 (26.1%)	13 (27.7%)	30 (27.8%)	0.997 ^[1]^
Low	30 (71.4%)	17 (73.9%)	34 (72.3%)	78 (72.2%)	
**Poverty Rate (%)**					
High	15 (35.7%)	8 (34.8%)	18 (38.3%)	22 (20.4%)	0.065 ^[1]^
Low	27 (64.3%)	15 (65.2%)	29 (61.7%)	86 (79.6%)	
**Food Stamps (%)**					
High	11 (26.2%)	3 (13.0%)	11 (23.4%)	21 (19.4%)	0.596 ^[1]^
Low	31 (73.8%)	20 (87.0%)	36 (76.6%)	87 (80.6%)	
**Proximity to Superfund Sites**					
High	6 (14.3%)	2 (8.7%)	8 (17.0%)	29 (26.9%)	0.112 ^[1]^
Low	36 (85.7%)	21 (91.3%)	39 (83.0%)	79 (73.1%)	
**Social Vulnerability Index (%)**					
High	16 (38.1%)	7 (30.4%)	11 (23.4%)	17 (15.7%)	0.026 ^[1]^
Low	26 (61.9%)	16 (69.6%)	36 (76.6%)	91 (84.3%)	
**Cigarette Smoking Rate (%)**					
High	15 (35.7%)	6 (26.1%)	14 (29.8%)	21 (19.4%)	0.183 ^[1]^
Low	27 (64.3%)	17 (73.9%)	33 (70.2%)	87 (80.6%)	
**Traffic Intensity**					
High	14 (33.3%)	3 (13.0%)	12 (25.5%)	27 (25.0%)	0.354 ^[1]^
Low	28 (66.7%)	20 (87.0%)	35 (74.5%)	81 (75.0%)	
**Proximity to Hazardous Waste Management Sites**					
High	20 (47.6%)	5 (21.7%)	16 (34.0%)	34 (31.5%)	0.15 ^[1]^
Low	22 (52.4%)	18 (78.3%)	31 (66.0%)	74 (68.5%)	
**Unemployment Rate (%)**					
High	12 (28.6%)	3 (13.0%)	13 (27.7%)	29 (26.9%)	0.52 ^[1]^
Low	30 (71.4%)	20 (87.0%)	34 (72.3%)	79 (73.1%)	
**Uninsured Rate (%)**					
High	12 (28.6%)	5 (21.7%)	12 (25.5%)	20 (18.5%)	0.545 ^[1]^
Low	30 (71.4%)	18 (78.3%)	35 (74.5%)	88 (81.5%)	
**Proximity to Water Polluting Sites**					
High	6 (14.3%)	3 (13.0%)	11 (23.4%)	34 (31.5%)	0.076 ^[1]^
Low	36 (85.7%)	20 (87.0%)	36 (76.6%)	74 (68.5%)	
**Lung Cancer Diagnosis Rate (per 100,000)**					
High	14 (33.3%)	6 (26.1%)	9 (19.1%)	21 (19.4%)	0.279 ^[1]^
Low	28 (66.7%)	17 (73.9%)	38 (80.9%)	87 (80.6%)	

[1]: χ^2^ test.

**Table 4:** Cox proportional hazards regression analysis of individual-level survival predictors. Model optimized via backward selection (C-index = 0.76).

Predictors	Hazard Ratio	95% CI	*p*
Stage (Ordinal)	1.70	1.34–2.17	**<0.001**
DDR Mutation: DDR +	Reference		
DDR Mutation: DDR–	1.16	0.72–1.87	0.553
Age: ≥60	Reference		
Age: <60	2.85	1.53–5.31	**0.001**
Insurance: Private Insurance	Reference		
Insurance: Medicaid	1.02	0.46–2.26	0.960
Insurance: Medicare	1.55	0.69–3.49	0.290
Insurance: Others	7.22	1.64–31.76	**0.009**
Tobacco: Never	Reference		
Tobacco: Former	2.30	1.02–5.19	**0.044**
Tobacco: Active	3.89	1.61–9.40	**0.003**
Cancer Type: Squamous Cell Carcinoma	Reference		
Cancer Type: Small Cell Carcinoma	1.37	0.38–4.97	0.632
Cancer Type: Adenocarcinoma	1.05	0.55–2.03	0.876
Cancer Type: Others	3.15	1.30–7.61	**0.011**
Life Expectancy (years): Low	Reference		
Life Expectancy (years): High	2.04	1.28–3.24	**0.003**
Observations	220		

**Table 5:** Predictors of survival: Community-level factors. Model optimized via backward selection (C-index = 0.78).

Predictors	Hazard Ratio	95% CI	*p*
Food Insecurity (%): High	Reference		
Food Insecurity (%): Low	2.33	1.35–4.03	**0.002**
Uninsured Rate (%): High	Reference		
Uninsured Rate (%): Low	2.39	1.26–4.54	**0.008**
Proximity to Potential Chemical Accidents: Low	Reference		
Proximity to Potential Chemical Accidents: High	1.73	1.03–2.90	**0.037**
Observations	220		

## Data Availability

Supplemental Table S1. Glossary of patient-linked social determinants of health (SDOH) and cancer risk factors, as compiled by the University of Illinois Cancer Center Data Integration Shared Resource, otherwise no data would be available to deposit in a repository.

## Supplementary Materials

The following supporting information can be downloaded at: https://www.mdpi.com/article/10.3390/cancers18162606/s1, Supplemental Methods S1: Supplemental Table S1: Glossary describing each of the population-level social determinants of health (“Population level data at the zip code level_550 measures.xlsx”); Supplemental Table S2: Logistic regression analysis of predictors for DDR+ (DNA damage repair-positive) status; Supplemental Figure S1: Geographical distribution of patients categorized as DNA damage response-positive (DDR+). Home addresses were geocoded to the census tract level and mapped using a standardized coordinate reference system (WGS84). Each point represents the residential location of an individual patient included in the DDR+ analysis group; Supplemental Figure S2: Geographical distribution of patients categorized as DNA damage response-negative (DDR-). Home addresses were geocoded to the census tract level and mapped using a standardized coordinate reference system (WGS84). Each point represents the residential location of an individual patient included in the DDR-analysis group. Supplemental Figure S3: Comparison of tumor mutational burden (TMB) in DDR-mutant and DDR-wild-type cohorts; Supplemental Figure S4: Overall survival (OS) analysis in the DDR-mutated (DDRmt) cohort. Kaplan–Meier curves comparing OS between patients harboring a single DDR gene mutation versus those with ≥2 DDR mutations. No statistically significant difference in OS was observed between the two groups (*p* > 0.05).
