# Supplementary material for: The Association of Social and Biological Factors with Clinical Outcomes in Patients with Lung Cancer and DNA Damage Repair Pathway Mutations: A Single-Institution Experience
Source: Cancers (Basel). 2026 Aug 13;18(16):2606. doi: 10.3390/cancers18162606 (PMC13511018; doi:10.3390/cancers18162606)

Supplemental Methods S1

Patient data were retrospectively collected from the electronic medical record (EMR) system at the University of Illinois Hospital (UIH). The following variables were extracted and defined as follows:

- **Insurance:** Insurance providers were categorized into predefined groups:
  - **Medicare:** Includes traditional Medicare and Medicare Advantage plans;
  - **Medicaid:** State and federal Medicaid programs;
  - **Private:** All other commercial or private insurance plans;
  - **VA Insurance:** Coverage through the Department of Veterans Affairs;
  - **Self-Pay:** Patients without insurance coverage who are responsible for their medical bills.
- **Tobacco Use:** Patients were classified as "current," "past," or "never" smokers. In cases of intermittent cessation, if the last note indicated the patient had stopped smoking, they were categorized as "former";
- **Pack-Years:** Calculated using the Epic EMR "pack-year" feature;
- **Low-Dose CT (LDCT) Screening:** Documented as "yes" if the patient had received a low-dose screening chest CT prior to their lung cancer diagnosis, Incidental lung cancer findings on CT scans performed for other indications were not counted as positive LDCT screening;
- **Date of Diagnosis:** Defined as the date of pathology specimen collection confirming the cancer diagnosis;
- **Stage at Diagnosis:** The cancer stage at the time of diagnosis;
- **Family History:** History of lung cancer in a first-degree relative.

Supplemental Table S1

Glossary describing each of the population level social determinants of health ("Population level data at the zip code level_550 measures.xlsx")

Supplemental Table S2: Logistic regression analysis of predictors for DDR+ (DNA damage repair-positive) status.

	**Backward Selection**		
Predictors	*Odds Ratios*	*CI*	*p*
(Intercept)	0.00	0.00 – 0.10	**0.040**
Area Deprivation Index	0.98	0.96 – 1.00	**0.040**
PM2.5 Concentration (μg/m3)	6.31	1.00 – 53.38	0.068
Squamous Cell Carcinoma	*Reference*		
Adenocarcinoma	10.03	2.89 – 63.42	**0.002**
Small Cell Carcinoma	4.75	0.50 – 46.18	0.153
Others	7.52	1.41 – 57.84	**0.026**
Observations	225		

Supplemental Figure S1: Geographical distribution of patients categorized as DNA damage response-positive (DDR+). Home addresses were geocoded to the census tract level and mapped using a standardized coordinate reference system (WGS84). Each point represents the residential location of an individual patient included in the DDR+ analysis group.

DDR POSITIVE PATIENTS

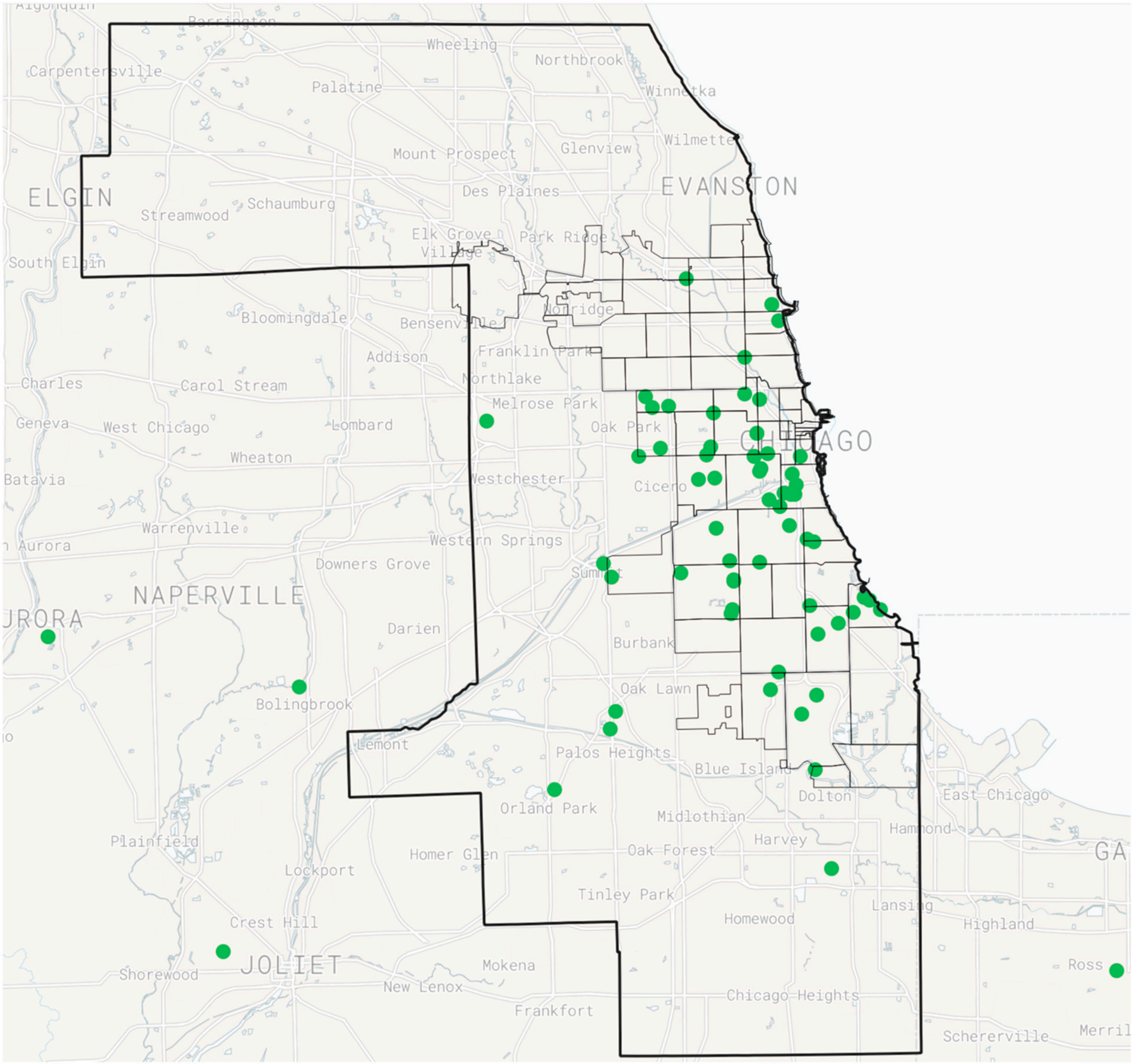

Supplemental Figure S2: Geographical distribution of patients categorized as DNA damage response-negative (DDR-). Home addresses were geocoded to the census tract level and mapped using a standardized coordinate reference system (WGS84). Each point represents the residential location of an individual patient included in the DDR- analysis group.

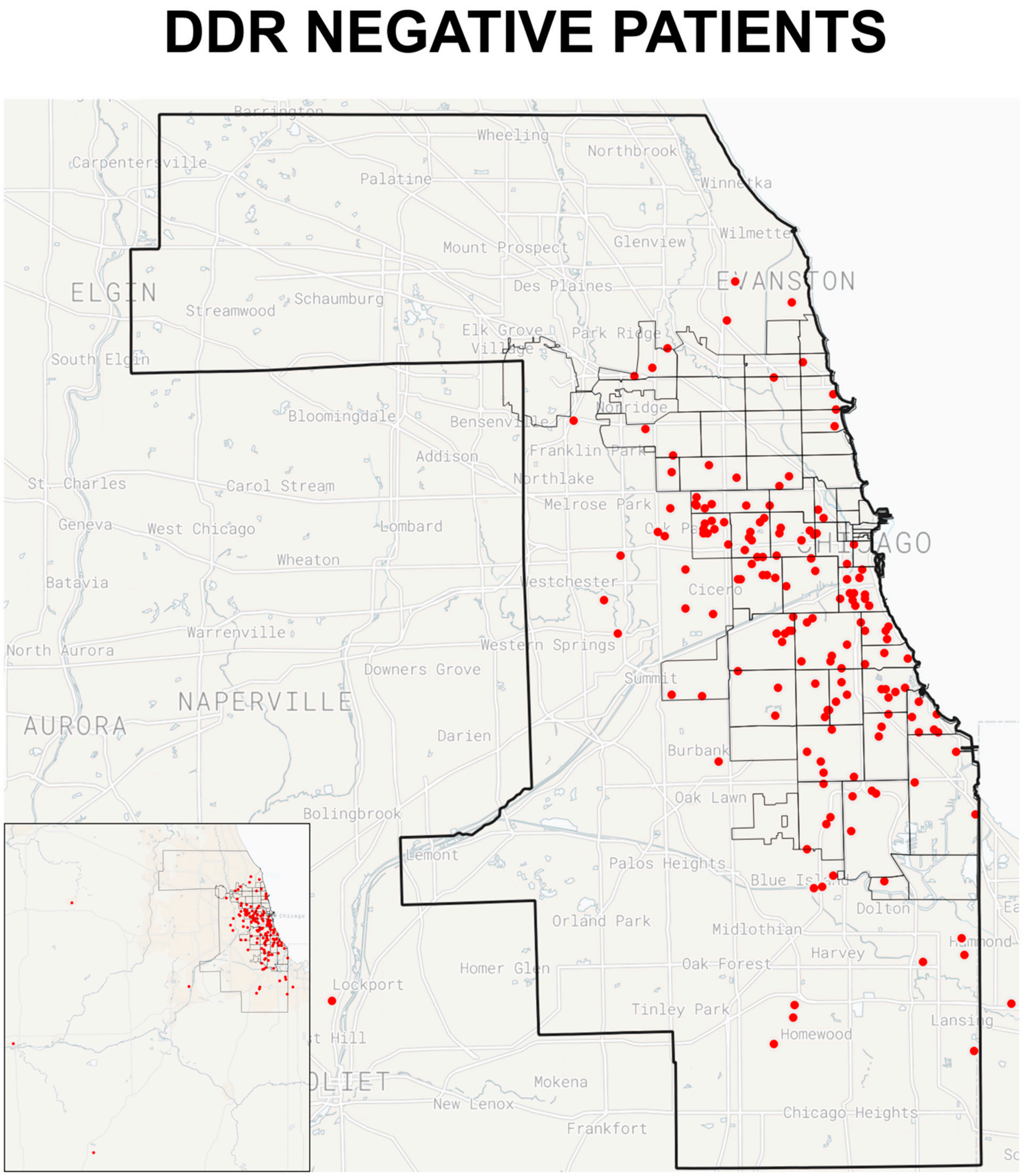

Supplemental Figure S3. Comparison of tumor mutational burden (TMB) in DDR-mutant and DDR-wild-type cohorts.

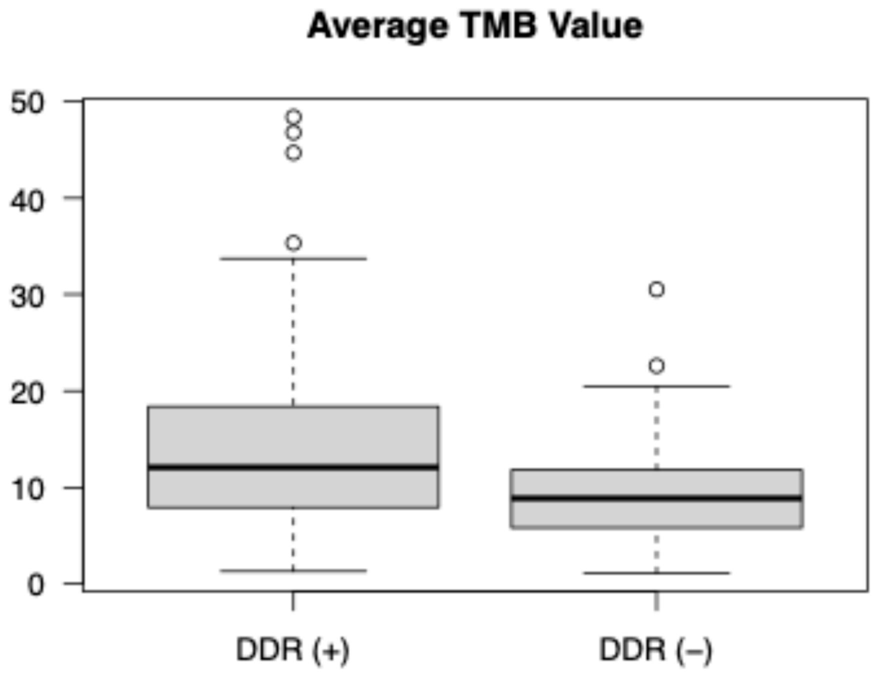

Supplemental Figure S4. Overall survival (OS) analysis in the DDR-mutated (DDRmt) **cohort.** Kaplan–Meier curves comparing OS between patients harboring a single DDR gene mutation versus those with ≥2 DDR mutations. No statistically significant difference in OS was observed between the two groups (p > 0.05).

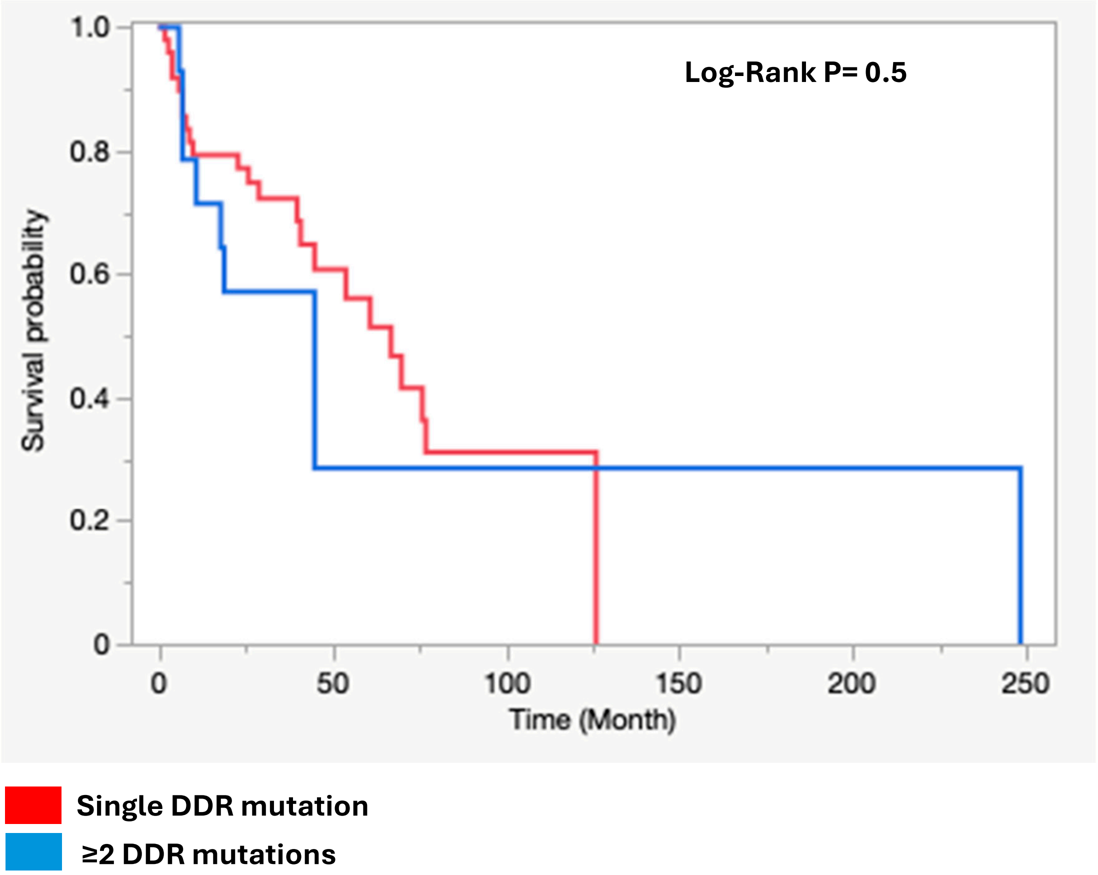

Supplement: Supplementary file 1 [file cancers-18-02606-s001.zip › cancers-4432588-supplementary.pdf]
